# Methamphetamine Self-Administration Is Associated with Persistent Biochemical Alterations in Striatal and Cortical Dopaminergic Terminals in the Rat

**DOI:** 10.1371/journal.pone.0008790

**Published:** 2010-01-20

**Authors:** Irina N. Krasnova, Zuzana Justinova, Bruce Ladenheim, Subramaniam Jayanthi, Michael T. McCoy, Chanel Barnes, John E. Warner, Steven R. Goldberg, Jean Lud Cadet

**Affiliations:** 1 National Institute on Drug Abuse, National Institutes of Health, Baltimore, Maryland, United States of America; 2 Department of Psychiatry, University of Maryland School of Medicine, Baltimore, Maryland, United States of America; INSERM U862, France

## Abstract

Methamphetamine (meth) is an illicit psychostimulant that is abused throughout the world. Repeated passive injections of the drug given in a single day or over a few days cause significant and long-term depletion of dopamine and serotonin in the mammalian brain. Because meth self-administration may better mimic some aspects of human drug-taking behaviors, we examined to what extent this pattern of drug treatment might also result in damage to monoaminergic systems in the brain. Rats were allowed to intravenously self-administer meth (yoked control rats received vehicle) 15 hours per day for 8 days before being euthanized at either 24 hours or at 7 and 14 days after cessation of drug taking. Meth self-administration by the rats was associated with a progressive escalation of daily drug intake to 14 mg/kg per day. Animals that self-administered meth exhibited dose-dependent decreases in striatal dopamine levels during the period of observation. In addition, there were significant reductions in the levels of striatal dopamine transporter and tyrosine hydroxylase proteins. There were also significant decreases in the levels of dopamine, dopamine transporter, and tyrosine hydroxylase in the cortex. In contrast, meth self-administration caused only transient decreases in norepinephrine and serotonin levels in the two brain regions, with these values returning to normal at seven days after cessation of drug taking. Importantly, meth self-administration was associated with significant dose-dependent increases in glial fibrillary acidic protein in both striatum and cortex, with these changes being of greater magnitude in the striatum. These results suggest that meth self-administration by rats is associated with long-term biochemical changes that are reminiscent of those observed in post-mortem brain tissues of chronic meth abusers.

## Introduction

Methamphetamine (METH) is a highly addictive psychostimulant drug whose abuse has reached epidemic proportions in the USA and worldwide [1]–[4]. This presents a serious public concern because chronic METH abuse is associated with major health problems including anxiety, depression, psychosis and psychomotor dysfunctions in humans [5], [6]. Cognitive studies of chronic METH users have also found deficits consistent with impaired functions of striatal and cortical systems. These include deficits in attention, learning, working memory, and decision making [7]–[11]. The accumulated evidence is compelling that the negative neuropsychiatric consequences of METH abuse are related to drug-induced pathological changes in the brains of METH addicts [12]. Specifically, clinical imaging studies have demonstrated significant reductions in the levels of dopamine transporters (DAT) [8], [11], [13] and reactive gliosis [14], [15] in the brains of METH abusers. Postmortem analyses of brain tissues obtained from METH addicts revealed significant decreases in dopamine (DA) concentrations and in DAT and tyrosine hydroxylase (TH) protein levels [16]–[18]. There is also neuroimaging evidence that these patients suffer from a marked loss of serotonin transporters (5-HTT) in several brain regions [19], although a recent post-mortem report showed that the reductions in 5-HTT might not be as widespread as suggested by the positron emission tomography data [20].

Animal studies have also provided extensive evidence for METH-induced damage to dopaminergic and serotoninergic terminals in rodent and nonhuman primate models using various drug dosing paradigms [21]–[23]. These include single large doses (30–100 mg/kg) or multiple injections of moderate doses (5–25 mg/kg) of the drug given in one day or over several days to assess the effects of METH on mammalian monoaminergic systems [24]–[28]. Because most rodent experiments involved multiple METH injections, given in a single day, in order to assess the effects of METH on brain monoaminergic systems, it has been suggested that this approach might not necessarily represent drug-taking behavioral patterns exhibited by METH addicts [29], [30]. For example, acute single-day binge injections do not replicate the progressive increases in the amount of METH taken by addicts over time as they develop tolerance to euphoric and other psychological effects of the drug [29], [30]. Because of these discrepancies, some groups have made attempts to mimic the human patterns of drug taking by passively increasing the doses of METH given to rodents [31]–[35]. Other investigators have used a METH self-administration paradigm [36]–[38] and have reported that animals given extended access to METH escalate their drug intake [36]–[38]. These observations have led to the suggestion that METH self-administration by rodents might represent a better model of human drug-taking behaviors [36]–[39]. Two groups of investigators, who have applied the self-administration approach using relatively small drug doses with either short (2 hours) [40] or more prolonged (9 hours) [41] sessions of daily METH exposure, have failed to find any evidence of significant neurotoxic damages in monoaminergic terminals in the rat brain. Of related interest, however, Schwendt at al. [42] have recently reported that extended (6 hours) access to METH self-administration caused persistent decreases in DAT protein expression in the prefrontal cortex and striatum without affecting DA and TH levels in these brain regions. Because the relative lack of biochemical abnormalities in these reports is not consistent with post-mortem findings in the brains of human addicts [16]–[18], more efforts are needed to clarify the divergent results and to better replicate the neurochemical data observed in the brains of METH addicts. This is of translational relevance because therapeutic approaches using the self-administration models need to better approximate the pathological substrates of METH addicted brains. We reasoned that larger doses of the drug self-administered over longer periods of time might be more relevant to human conditions because METH addicts are known to spend hours injecting METH over several days [29], [30]. Here we present data showing that extended (15 hours a day) access to METH self-administration over eight consecutive days which is associated with increasing METH intake by rats does indeed result in significant decreases of striatal and cortical DA levels measured up to 14 days after the last drug session. This pattern of METH administration is also associated with decreases in the expression of DAT and TH proteins in these brain regions. Moreover, METH caused increases in the expression of GFAP, a well-known marker of toxic insults in the brain [43].

## Results

### METH Self-Administration and Intake

**Figure 1:**
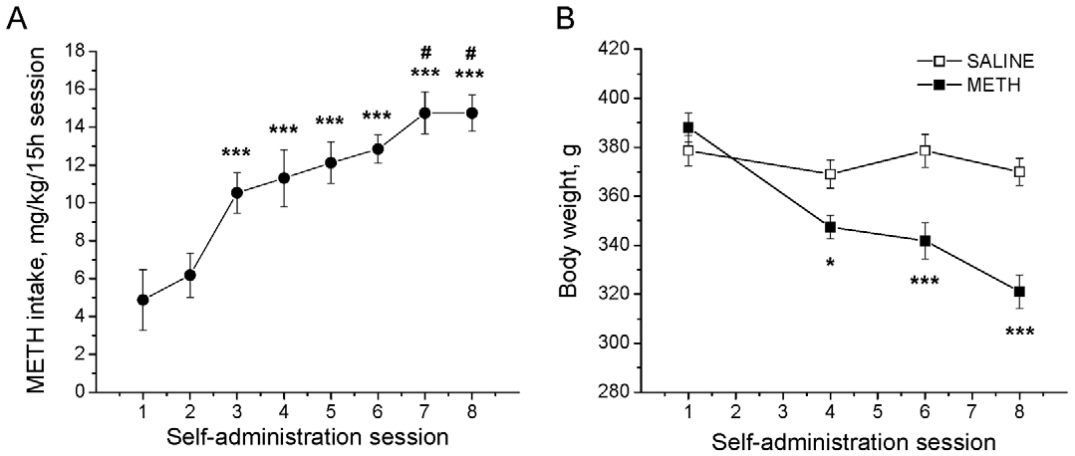
Changes in METH intake and body weight over the course of self-administration in rats. (A) Escalation of METH intake in rats. Symbols indicate average daily amount of actively self-administered METH during each of 8 consecutive daily sessions (means ± SEM; n = 11). Rats increased METH intake on days 3–8 compared with days 1 and 2, and days 7–8 showed further increases in intake compared with day 3. Data analyzed by one-way ANOVA for repeated measures, followed by Tukey's multiple comparison test: *** *p*<0.001 in comparison with sessions 1 and 2, # *p*<0.05 in comparison with session 3. (B) METH caused significant decreases in body weight in the animals that actively self-administered the drug (means ± SEM; n = 9). Data analyzed by two-way ANOVA for repeated measures, followed by Tukey's multiple comparison test: * *p*<0.05; *** *p*<0.001 in comparison to saline group.

We used an extended access model of intravenous METH self-administration to investigate the effects of voluntary METH intake on monoaminergic systems in the brain. Rats were allowed to self-administer METH (0.1 mg/kg/injection, i.v.) during daily 15-hour sessions for 8 consecutive days. Animals in a yoked control group received injections of saline whenever rats in the test group self-administered METH. Fig. 1A shows that the average daily METH intake during each 15-hour daily session in rats gradually increased starting by the third session (F_(7,70)_ = 20.004; *p*<0.001). These increases lasted throughout the eighth session, as compared with the first (*p*<0.001) and second sessions (*p*<0.001). Further escalation of drug taking was manifested by significantly higher METH intake during the seventh session when compared with the third session (*p*<0.05). Self-administration of METH at this level was maintained during the eighth session. The average daily intake reached 14.8±0.8, 14.0±1.6 and 14.7±1.1 mg/kg/day for three groups of animals euthanized at 24 hours, 7 and 14 days, respectively, after the last METH self-administration session. Total cumulative drug intake over 8 sessions was 89.1±7.1, 81.2±11.9 and 87.4±7.2 mg/kg for animals euthanized at 24 hours, 7 and 14 days, respectively, after the last drug session. There were no significant differences in average daily METH intake or total amount of drug taken between the groups.

### METH-Induced Changes in Body Weight

Fig. 1B shows the effects of METH self-administration on body weight in rats. There were significant decreases in body weights in the group of METH-taking rats, with the animals losing about 17% of their weights by the eighth self-administration session. The yoked saline-receiving group maintained stable weights throughout the experiment. The difference in body weights between the two groups was significant starting by the fourth session (F_(3,48)_ = 69.62; *p*<0.001). The observation of weight loss in the METH group is consistent with the report by Davidson *et al.* [44] who also found that rats treated with METH via mini-pumps lost weight from day 2 onwards during a 7-day drug administration schedule.

### METH-Induced Changes in Monoamine Levels in the Rat Brain

#### Striatum

**Figure 2:**
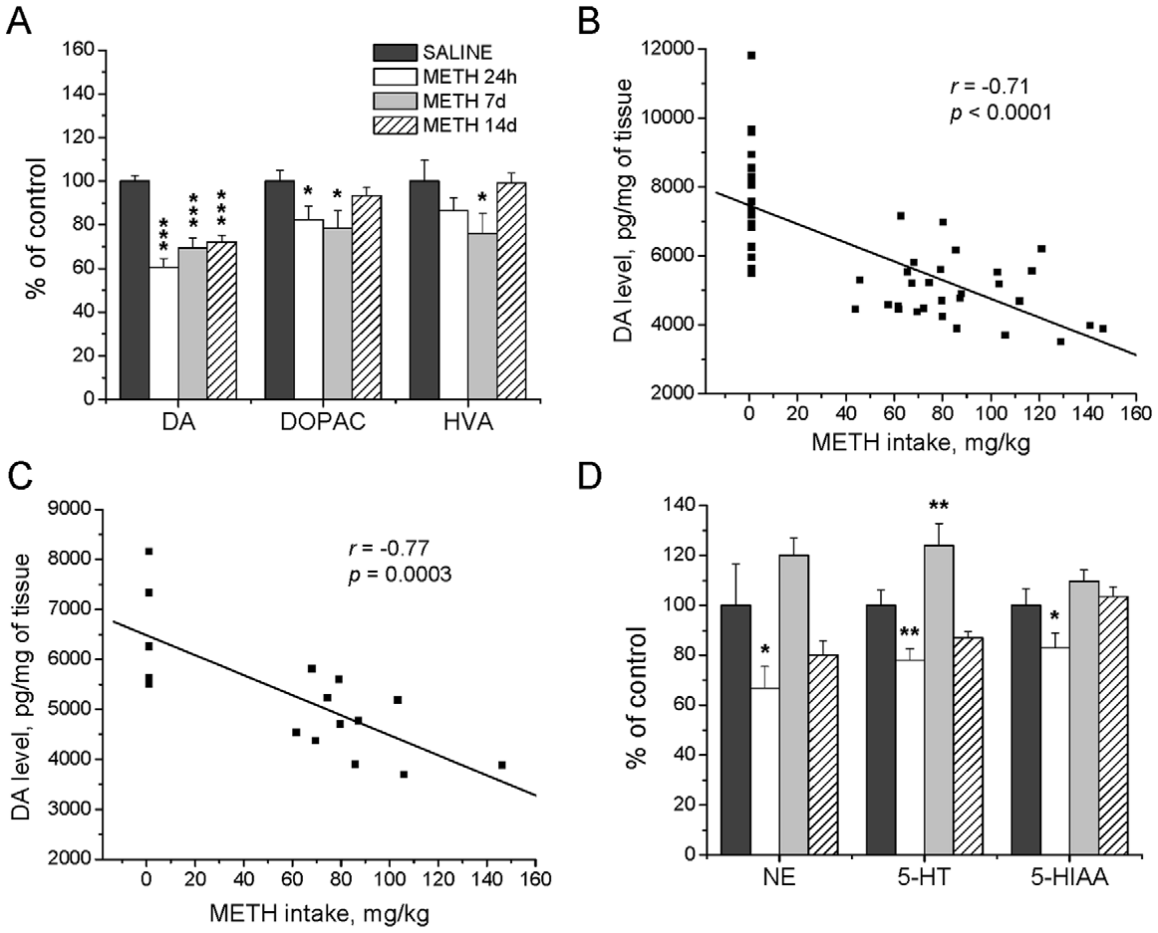
Effects of METH self-administration on monoamine levels in the striatum. METH caused persistent decreases in striatal DA levels (A), which negatively correlated with total drug intake when data at 24 hours, 7 and 14 days after cessation of self-administration were combined (B) and at 14 days post-drug (C). However, only transient changes were found in the levels of NE and 5-HT (D). Data shown as mean ± SEM. *, *p*<0.05; **, *p*<0.01; ***, *p*<0.001 vs control. Data were analyzed by ANOVA followed by PLSD, n = 7−11 per group. Correlation analysis was done by regression analysis.

To investigate whether METH self-administration causes deleterious effects on monoaminergic terminals, we measured the levels of monoamines in the striatum. METH induced significant and similar decreases in DA levels in animals euthanized at 24 hours (−39%), at 7 (−31%) and at 14 days (−28%) after cessation of METH self-administration (F_(5, 47)_ = 12.91; *p*<0.0001) (Fig. 2A). 3,4-dihydroxyindoleacetic acid (DOPAC) levels were also significantly affected by METH at 24 hours (−18%) and 7 days (−21%) (F_(5,47)_ = 2.49; *p* = 0.044), while homovanillic acid (HVA) levels were decreased only at the 7-day time point (−24%) (F_(5,47)_ = 2.70; *p* = 0.042). In order to assess if there was a relationship between the dose of METH and striatal DA depletion, we used regression analysis on the combined dataset of the three time-points and found a significant negative correlation between METH intake by individual rats and striatal DA levels (Pearson's correlation; *r* = −0.71, *r^2^* = 0.50, *p*<0.0001) (Fig. 2B). A similar negative correlation (*r* = −0.77, *r^2^* = 0.59, *p* = 0.0003) was also observed when data for only the 14-day time-point were considered (Fig. 2C).

In addition to the effects on the nigrostriatal dopaminergic system, METH self-administration was associated with significant decreases in norephinephrine (NE) (−33%) (F_(5,47)_ = 4.11; *p* = 0.004), serotonin (5-HT) (−22%) (F_(5,47)_ = 7.56; *p*<0.0001) and 5-hydroxyindole acetic acid (5-HIAA) (−17%) (F_(5,47)_ = 3.05; *p* = 0.018) levels, which were present only at the 24-hour time point (Fig. 2D). Unexpectedly, the levels of striatal 5-HT were somewhat higher (+24%) than control values at the 7-day time-point but were similar to control levels at 14 days after cessation of METH self-administration.

#### Cortex

**Figure 3:**
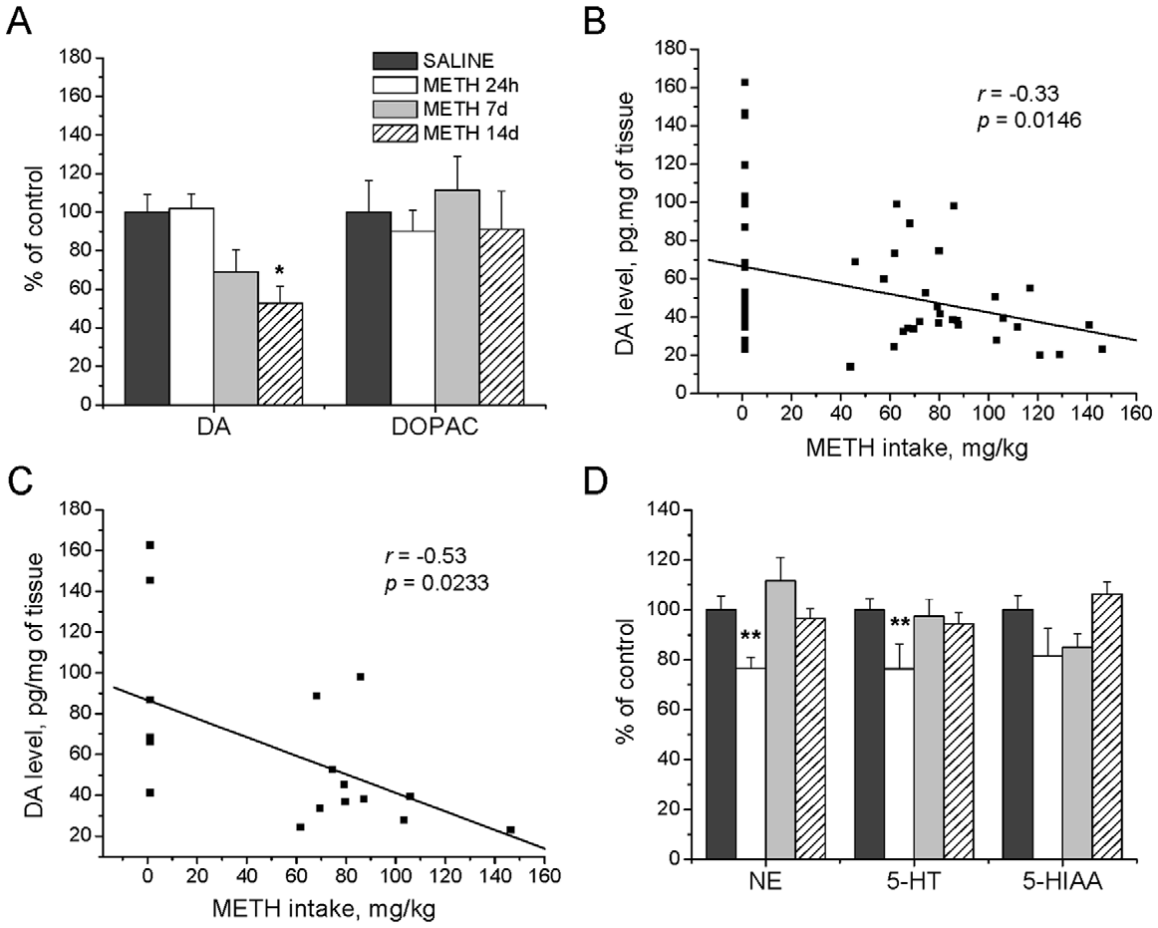
Effects of METH self-administration on monoamine levels in the cortex. METH self-administration resulted in long-term reductions in DA concentrations in the cortex (A). DA levels showed negative correlations with total METH intake at the 24 hour, 7 and 14 days time-points (B) and at 14 days after cessation of self-administration (C). METH caused only transient reductions in NE and 5-HT concentrations in the cortex (D). Data shown as mean ± SEM. *, *p*<0.05; **, *p*<0.01 vs control. Data were analyzed by ANOVA followed by PLSD, n = 7−11 per group. Correlation analysis was done by regression analysis.

Because binge METH treatment is also known to affect cortical monoaminergic systems [25], [26], [45]–[47], we examined the effects of METH self-administration in the cortex. In contrast to the striatal data, METH self-administration was associated with more gradual decreases in DA levels in the frontal cortex which reached significance at 14 days post-drug (−47%) (F_(5,47)_ = 3.13; *p* = 0.016) (Fig. 3A). DOPAC concentrations were not affected (Fig. 3A). Similar to the observations in the striatum, cortical DA levels correlated negatively with the doses of METH taken by the animals when data were combined for all three time-points (*r* = −0.33; *r^2^* = 0.11; *p* = 0.0146) (Fig. 3B). Similar results were obtained for the 14-day time-point (*r* = −0.53; *r^2^* = 0.28; *p* = 0.0233) (Fig. 3C).

Animals that self-administered METH exhibited decreases in NE levels in the frontal cortex at 24 hours (−24%) (F_(5,47)_ = 3.89; p = 0.005) after cessation of drug intake but not at other times (Fig. 3D). There were also METH-induced decreases in 5-HT levels only at the 24-hour time-point (−24%) (F_(5,47)_ = 2.45, *p* = 0.047) but no significant changes in 5-HIAA levels (Fig. 3D).

### Effects of METH Self-Administration on TH, DAT, and 5-HTT Protein Levels in the Striatum and Cortex

**Figure 4:**
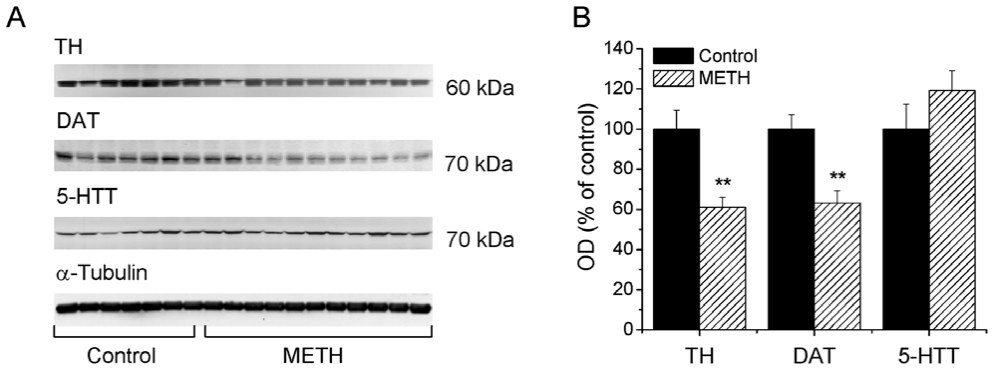
METH self-administration causes reductions in DAT and TH protein levels in the striatum. (A) Representative immunoblots show expression of TH, DAT and 5-HTT protein levels in the striatum 14 days after last METH self-administration session. (B) Quantitative analyses of immunoblots reveal significant decreases in TH and DAT, but no change in 5-HTT expression in the METH-treated rats. Data presented as mean ± SEM. ** *p*<0.01 vs control. Data were analyzed by ANOVA followed by PLSD, n = 7−11 per group.

**Figure 5:**
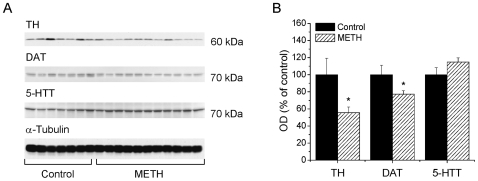
METH self-administration resulted in decreased expression of DAT and TH proteins in the cortex. (A) Immunoblots showing TH, DAT and 5-HTT protein expression in the cortex 14 days after cessation of METH intake. (B) Quantification of METH toxic effects demonstrates significant decreases in TH and DAT, but not in 5-HTT protein levels. Data shown as mean ± SEM. * *p*<0.05; ** *p*<0.01 vs control. Data were analyzed by ANOVA followed by PLSD, n = 7−11 per group.

Because the accumulated evidence has suggested that toxic doses of METH can cause damage to monoaminergic terminals in the rodent brain [23], we used Western blot analysis to measure levels of TH, DAT and 5-HTT, markers of DA and 5-HT terminal integrity, in the striata and cortices of animals euthanized at 14-day time-point. Fig. 4A shows the results of Western blots in the striatum while Fig. 4B presents the quantitative data obtained from these experiments. METH self-administration caused significant decreases in striatal TH (−39%) (F_(1,16)_ = 12.84; *p* = 0.003) and DAT (−37%) (F_(1,16)_ = 14.24; *p* = 0.002) protein levels. However, there were no significant changes in the expression of 5-HTT protein (Fig. 4B). Similar to the striatum, METH self-administration induced significant decreases in the levels of cortical TH (−45%) (F_(1,16)_ = 6.41; *p* = 0.022) and DAT (−23%) (F_(1,16)_ = 5.11, *p* = 0.040) without affecting 5-HTT protein expression (Fig. 5A and B).

### Effects of METH Self-Administration on GFAP Levels in the Striatum and Cortex

**Figure 6:**
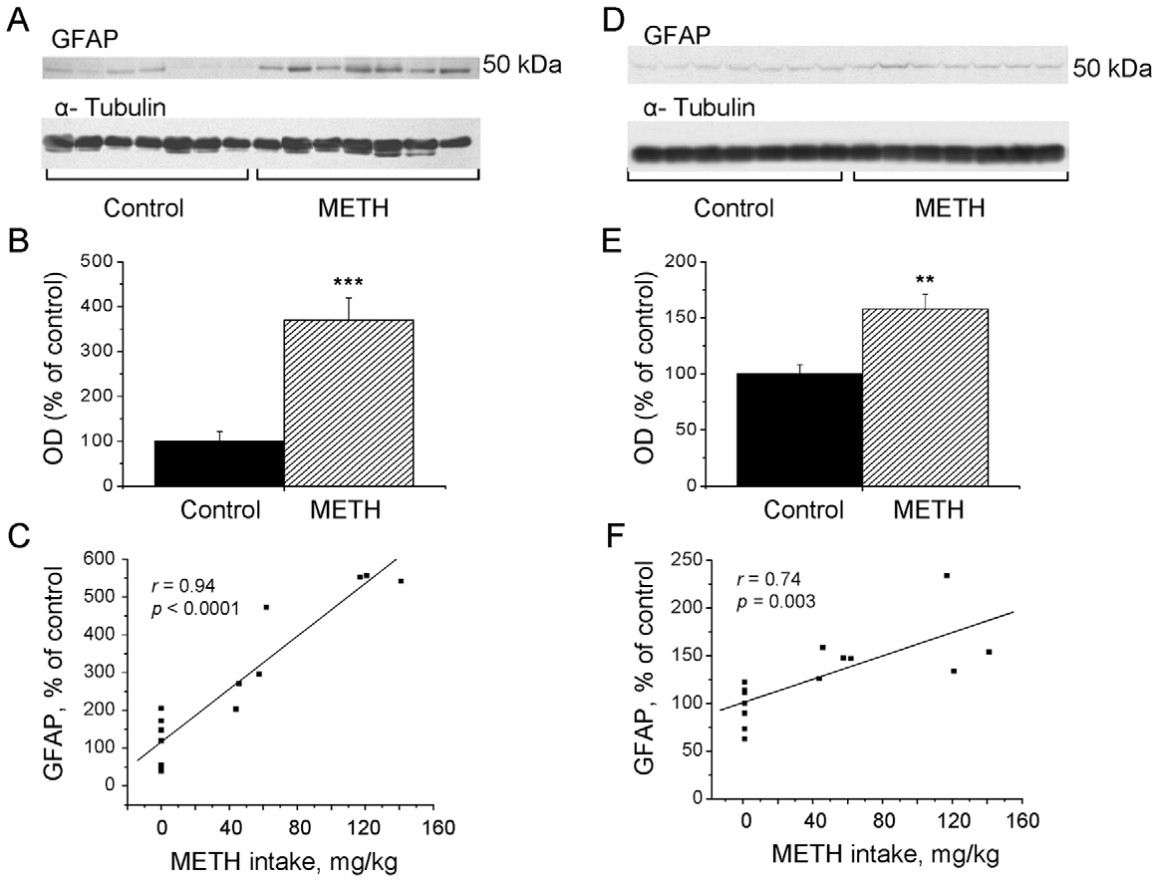
Effects of METH self-administration on GFAP expression in the striatum and cortex. Representative Western blot analyses show that the METH self-administering rats had increased GFAP levels in their striata (A) and cortices (D). Quantitative analyses of the Western blots show significant increases in GFAP levels in the METH-treated rats (B, E). Key to statistics: ** *p*<0.01, *** *p*<0.001 (ANOVA, n = 7 per group). GFAP expression showed significant positive correlation with METH intake in the striatum (C) and cortex (F).

Toxic doses of METH are also known to cause reactive astrocytosis in the mammalian brain [48], [49]. Therefore, we used Western blot analysis to measure levels of GFAP in the striata and cortices of animals euthanized at the 7-day time-point. The results of the Western blots in the striatum are shown in Fig. 6A and the quantitative data are presented in Fig. 6B. METH self-administration caused marked increases in striatal GFAP levels (+370%) (F_(1,12)_ = 23.131; *p* = 0.0004), which correlated positively with the doses of METH self-administered by the animals (*r* = 0.94; *r^2^* = 0.88; *p*<0.0001) (Fig. 6C). There were also significant METH-induced increases in GFAP levels in the cortex (+58%) (F_(1,12)_ = 15.042; *p* = 0.0019) (Fig. 6D and E). Similar to the striatal observations, there was a positive correlation between GFAP expression in the cortex and total METH intake by individual animals (*r* = 0.74; *r^2^* = 0.55; *p* = 0.003) (Fig. 6F).

## Discussion

The present experiments demonstrated that (1) extended access to METH self-administration was associated with a progressive escalation of drug intake by rats and significant decreases in their body weights; (2) this pattern of METH intake resulted in persistent dose-dependent depletion of striatal and cortical DA levels measured at various times after cessation of drug treatment; (3) METH self-administration also caused decreases in the expression of striatal and cortical TH and DAT proteins; and (4) METH induced dose-dependent increases in GFAP expression in both, striatum and cortex.

Our results are in agreement with findings from post-mortem studies that have reported decreased DA levels and reduced TH and DAT protein expression in the striata of human METH addicts [16]–[18]. They are also consistent with animal studies showing that METH treatment can cause persistent abnormalities in the markers of DA system integrity in various brain regions [25]–[27], [45], [50]. Our findings are also in agreement with clinical data demonstrating reactive astrocytosis in the brains of chronic METH abusers [14] and with observations of reactive astrocytosis in rodent striatum following METH injections [48], [49], [51]. However, our results are different from those of Shepard et al. [41] and Stefanski et al. [40] who reported that METH self-administration did not induce persistent changes in markers of DA system integrity in the rat brain. Yet, they are in partial agreement with data of Schwendt et al. [42], who showed very recently that METH self-administration caused decreases in the expression of DAT protein in the striatum and cortex without affecting DA concentrations or TH protein levels. Possible reasons for the differences include the use of lower doses of self-administered METH and the shorter daily drug access allowed to rats in these studies. Specifically, Stefanski *et al.* [40] reported that rats self-administered 2.5 mg/kg/day of METH over a 2-hour daily session, Schwendt et al. [42] stated that rats self-administered about 7 mg/kg/day during 6-hour sessions, while Shepard *et al.* [41] found that their animals took 7.9 mg/kg/day of METH during 9 hours of access. In the present study, rats reached an average METH intake of 14 mg/kg/day during the 15-hour sessions. Together, these findings suggest that METH can cause both neuroadaptative and neurotoxic changes in the mammalian brain depending on the intake, with lower doses of METH being able to induce transient alterations in markers of DA system integrity [40], [41] or neuroadaptive decreases in DAT protein expression without affecting DA and TH levels in the brain [42]. In contrast, higher METH doses can cause persistent abnormalities in DA, DAT, and TH levels that can last weeks and months after cessation of drug administration [25], [35], [45], [50]. The idea that METH causes dose-dependent alterations in the brain is supported by the present observations of negative correlation between DA levels in the striatum and cortex and the quantity of METH self-administered by individual rats. This discussion is further supported by the reports of positive correlation between METH intake and cell death in the prefrontal cortex [37] but negative correlation between METH intake and hippocampal volume [39] in rodents.

The mechanisms underlying METH-induced neurotoxic effects are thought to involve the production of reactive oxygen (ROS) and reactive nitrogen species (RNS) which overwhelm antioxidant defense systems in the brain [21], [23]. ROS and RNS can damage macromolecules such as DNA, proteins, and lipids causing oxidative stress [52]. Although neurons are thought to be very susceptible to oxidative injury [53], [54], oxidative stress can also trigger cellular and molecular responses that lead to activation of glia-mediated events which might cause additional damage to the brain [55]–[57]. Similar mechanisms appear to participate in the degeneration of the nigrostriatal DA pathway that occurs in Parkinsonism [57], [58]. Thus, the present observations showing greater magnitude of METH-induced reactive astrocytosis in the striatum than in the cortex might be responsible, in part, for the more severe abnormalities observed in that brain region. This might occur through the production of proinflammatory cytokines and chemokines by reactive glia [56], [58]. It is, nevertheless, possible that trophic factors secreted by astrocytes [59], [60] might also play role in the delayed partial recovery that occurs in the brain of rodents treated with toxic doses of METH [23], [25], [45].

Extending the duration of daily self-administration sessions to 15 hours resulted in an escalating increase in METH intake similar to reported in previous studies that used extended access to the drug [36]–[38], [42]. Because METH intake was also reported to escalate in human abusers during development of METH dependence [29], [30], the present model appears to better replicate some aspects of the human condition. The quantity of METH intake (about 14 mg/kg/day) reached by rats in our study is within the range of doses used by some human abusers who inject comparatively large doses of METH (11.7–32 mg/kg/day) during runs and binges [29], [30]. An important consequence of the large METH doses administered by these rats was the loss of body weight. This physiological complication is consistent with significant anorexia and weight loss reported by human abusers [30], [61], [62]. In fact, amphetamines have been used and abused in the self-treatment of obesity [62]–[64].

The METH-induced weight losses observed in the present study are consistent with the results reported by Bittner *et al.* [65] who found decreases in body weight in rats treated with a high-dose METH regimen (50 mg/kg given twice a day for 4 days). These METH doses also caused striatal DA depletion [65]. Moreover, Davidson *et al.* [44] also showed that treatment of rats with 7-day METH by minipumps (20 mg/kg/day) caused weight loss from day 2 onwards after implantation of the pumps. Although our results are in contrast to the report of Shepard *et al.* [41] who found that rats which self-administered lower doses of METH did not lose weight, the accumulated evidence suggests that METH can have significant anorectic effects [62]–[64]. Thus, it is of interest that Barsdley and Bachelard [66] reported that rats treated chronically with METH experienced decreases in TH enzyme activity in their brains only if they continued to experience the anorectic effects of the drug. Tolerant animals did not show any METH-induced biochemical deficits [66]. Therefore, when taken together with findings of significant weight loss in the present study, all these observations suggest that resistance to METH-induced anorexia during self-administration might predict, to a certain degree, tolerance to neurotoxic effects of METH.

In summary, this is the first demonstration that the striatal and cortical dopaminergic systems of rats can sustain damage from METH self-administration when animals are given extended access to higher doses of the drug, in a manner comparable to experienced human addicts who are known to abuse large quantities of intravenous METH during runs and binges [29], [30]. Because of the similarities between our results and those of human post-mortem studies that have recorded substantial depletion of DA levels and decreases in the expression of TH and DAT proteins in the brains of METH addicts [16]–[18], it is possible to suggest that rodent models aimed at replicating these biochemical deficits should allow for longer periods of daily access to the drug during self-administration experiments. Finally, because the present model also replicates patterns of METH abuse by humans, it might serve to better inform translational developments of therapeutic approaches to METH addiction.

## Materials and Methods

### METH Self-Administration

#### Subjects

Male Sprague-Dawley rats (Charles River, Wilmington, MA), weighing approximately 350–420 g at the beginning of the self-administration experiment, were individually housed in a temperature- and humidity-controlled environment under a reversed lighting 12-h light/dark cycle (lights on at 7:00 p.m.). The rats were allowed free access to food (NIH07 biscuits) in their home cage throughout the study. Water was available *ad libitum* in the home cage and in the testing chamber. Rats were tested in the light phase. They were experimentally and drug naïve at the beginning of this study.

Animals were maintained in facilities fully accredited by the American Association for the Accreditation of Laboratory Animals and all experiments were conducted in accordance with the guidelines of the Institutional Care and Use Committee of the Intramural Research Program, NIDA, NIH, and the Guidelines for the Care and Use of Mammals in Neuroscience and Behavioral Research (National Research Council 2003).

#### Apparatus

Eighteen standard operant-conditioning chambers (Coulbourn Instruments, Lehigh Valley, PA) were used. Each chamber contained a white house light and two holes with nose-poke operanda on either side of a food trough. Each nose poke produced a brief feedback tone. One hole was defined as active (left in nine chambers, right in remaining nine) and the other hole as inactive. METH or saline were delivered through Tygon tubing, protected by a metal spring and suspended through the ceiling of the experimental chamber from a single-channel fluid swivel. The tubing was attached to a syringe pump (Harvard Apparatus, South Natick, MA), which was programmed to deliver 2-s injections. The injected volume was adjusted for every animal to deliver a METH dose of 0.1 mg/kg/injection. Experimental events were controlled by microcomputers using MED Associates interfaces and software (Med Associates Inc., East Fairfield, VT).

Under anesthesia with a mixture of ketamine and xylazine (60 and 10 mg/kg i.p., respectively), rats were prepared with a silastic catheter implanted into the external jugular vein, with the catheter exiting the skin behind the ear. After catheter implantation, a nylon bolt glued to an acrylic mesh was implanted subcutaneously in the midscapular region. The nylon bolt served as a tether, preventing the catheter from being pulled out during self-administration sessions. Following surgery, the IV catheter was flushed daily during the first week with 0.2–0.3 ml of sterile 0.9% saline containing heparin (1.25 units/ml) and gentamicin (10 mg/ml) and then flushed after each daily session with heparin solution to maintain its patency.

#### Procedure

Each of the three experimental groups started with 18 naïve rats that were divided into two groups and tested simultaneously. One group served as yoked controls and passively received an injection of saline (which was not contingent on responding) each time a response-contingent injection of 0.1 mg/kg METH was actively self-administered by the first group of rats. Nose-poke responses by the yoked control rats were recorded, but had no programmed consequences. The first two experimental groups consisted of 9 rats self-administering METH and 9 yoked control rats. The third experimental group consisted of 11 rats self-administering METH and 7 yoked control rats.

15-hour sessions were conducted for 8 consecutive days, between 5 p.m. and 9 a.m. At the beginning of each session, a white house light was turned on and a priming injection of 0.1 mg/kg METH (or saline for yoked group), sufficient to fill the “dead” space of the IV catheter, was automatically delivered. Each nose-poke response in the active hole (fixed-ratio one, FR1) delivered an IV injection of 0.1 mg/kg of METH followed by 30-s timeout period, during which the chamber was dark and responses in either hole had no programmed consequences. Nose-poke responses in the “inactive” hole were recorded but had no programmed consequences. After eight sessions, rats were euthanized by decapitation. The first group of rats was euthanized at 24 hours, the second group at 7 days and the third group at 14 days after the last session ended. Brains were quickly removed and dissected on an iced plate.

### HPLC

For monoamine analysis, striata and cortices dissected from rat brains were homogenized in 0.01 M HClO_4_ and centrifuged at 18,000×g for 15 min. NE, DA, DOPAC, HVA, 5-HT and 5-HIAA levels were analyzed in brain tissue extracts using HPLC with electrochemical detector as previously described [67]. Monoamine levels were expressed as ng/mg of tissue weight and reported as % of control concentrations for ease of presentation.

### Western Blot

Western blot analyses were carried out as previously published [68]. In brief, striatal and cortical samples were washed with ice-cold 0.1 M PBS, homogenized in lysis buffer (0.01 M Tris-HCl, pH 7.6, 0.1 M NaCl, 0.001 M EDTA, 100 µg/ml PMSF and 1 µg/ml aprotinin) and then centrifuged at 15,000×g for 30 min. Protein concentration were determined with BioRad D_c_ Protein assay (BioRad, Temecula, CA). 10 µg of protein (striatum) and 40 µg of protein (cortex) were electrophoresed on 10% SDS-polyacrylamide gels and then transferred to Hybond-P™ membrane (GE Healthcare, Piscataway, NJ). The membranes were blocked and then immunolabeled with antibodies against DAT (1∶1000), TH (1∶4000), 5-HTT (1∶1000) and GFAP (1∶5000) (all from Millipore, Billerica, MA) at 4°C overnight. Immune complexes were detected with HRP-labeled second antibody and ECL+ chemiluminescence reagents (GE Healthcare). To confirm equal protein loading, blots were stripped and reprobed with α-tubulin antibody (1∶2000; Sigma, St. Louis, MO) for 2 hours at room temperature. Signal intensity was measured using densitometric analysis (UVP Inc., Upland, CA) and quantified using LabWorks analysis software (version 4.5).

### Statistical Analyses

All data are presented as means ± SEM. Statistical analysis of self-administration experimental data was performed using one-way analysis of variance (ANOVA) for repeated measures (intake) or two-way ANOVA for repeated measures (weights) followed by a pairwise multiple comparison procedure (Tukey's test) to identify differences between the sessions or groups, respectively (SigmaStat software: http://www.systat.com.) Statistical analysis of biochemical data was performed using ANOVA followed by Fisher's protected least significant difference (PLSD) (StatView 4.02, SAS Institute, Cary, NC). The relationship between METH intake, DA levels and GFAP expression was determined using Pearson's regression analysis (Origin 6.1, Origin Lab, Northampton, MA). The null hypothesis was rejected at *p*<0.05.
